# Supplementary material for: Characterization of an open-channel structure and lateral conduction pathway in the cation-selective pentameric ligand-gated ion channel, ELIC
Source: J Gen Physiol. 2026 Aug 27;158(5):e202513926. doi: 10.1085/jgp.202513926 (PMC13520879; doi:10.1085/jgp.202513926)
Supplement: Supplementary file 1 [file jgp_202513926_review_history.pdf]

Characterization of an Open-Channel Structure and Lateral Conduction Pathway in the Cation-Selective Pentameric Ligand-Gated Ion Channel, ELIC

Mark Arcario, Elizabeth Wu-Chen, Yuna Shim, Jérôme Hénin, Grace Brannigan, and Wayland Cheng

Corresponding Author(s): Wayland Cheng, Washington University in St. Louis

Review Timeline:

Submission Date:	November 7, 2025
Editorial Decision:	January 6, 2026
Revision Received:	July 3, 2026
Editorial Decision:	July 11, 2026
Revision Received:	July 20, 2026

Editor: Crina Nimigean

Transaction Report:

(Note: With the exception of the correction of typographical or spelling errors that could be a source of ambiguity, letters and reports are not edited. The original formatting of letters and referee reports may not be reflected in this compilation.)

DOI: https://doi.org/10.1085/jgp.202513926

January 6, 2026

Dr. Wayland W. L. Cheng
Washington University School of Medicine
Anesthesiology
660 S. Euclid Ave.
St. Louis, MO 63110

Re: 202513926

Dear Wayland,

Thank you for submitting your manuscript, entitled "Characterization of an Open-Channel Structure and Lateral Conduction Pathway in the Cation-Selective Pentameric Ligand-Gated Ion Channel, ELIC" to JGP. Your manuscript has now been seen by 3 reviewers, whose comments are appended below. You will see that the reviewers were very enthusiastic about the study and its potential impact but raised some concerns that should be addressed prior to further consideration of the manuscript at JGP. In particular, please view the editor's summary directly below.

Editor's Summary:

The proposed model relies heavily on a single 1 microsecond trajectory showing lateral pathway opening. Evidence that that this behavior is reproducible across independent simulations (which need not be of equal length), along with a brief mechanistic discussion of how this widening occurs, is needed. Furthermore, statistical rigor should be improved, including reporting conductance uncertainties across replicas, clarifying fitting procedures, and examining potential non-linear behavior by considering simulations at lower voltages.
Although larger lateral openings improve rectification, simulated conductances remain higher than experimental estimates. Additional analysis of this discrepancy is needed. Related, please discuss the potential issues caused by the lipid composition mismatch between simulations and experiments. In addition, the consequence of the use of harmonic restraints on the observed conductances should be clarified.
The manuscript could more clearly acknowledge the limitations of computational electrophysiology, including force-field approximations and lack of explicit electronic polarizability, and discuss whether the observed agreement with experiments may be partly fortuitous. In general, a more balanced framing of computational-experimental comparisons would be desirable and also strengthen the manuscript.

****

We hope that you will be able to submit a revised manuscript that addresses these points, which we believe will pose no problems, and which will be re-reviewed. In addition, please do not hesitate to contact me (via the editorial office) if you feel that a discussion of the reviewers' and editors' comments would be helpful.

Please submit your revised manuscript via the link below, along with a point-by-point letter that details your response to the reviewers' and editor's summary, as well as a copy of the text with alterations highlighted (boldfaced or underlined). If the article is eventually accepted, it would include a 'revised date' as well as submitted and accepted dates. If we do not receive the revised manuscript within one year, we will regard the article as having been withdrawn. We would be willing to receive a revision of the manuscript at a later time, but the manuscript will then be treated as a new submission, with a new manuscript number.

Please pay particular attention to recent changes to our instructions to authors in the following sections: Data presentation, Blinding and randomization and Statistical analysis, under Materials and Methods, as shown here: https://rupress.org/jgp/pages/submission-guidelines#prepare. Re-review will be contingent on inclusion of the required information (including for data added during revision) and demonstration of the experimental reproducibility of the results. Also, To improve the reproducibility of published content, we have partnered with SciScore. Authors are prompted in eJP to copy and paste the Materials and Methods section of their manuscript for a SciScore assessment when submitting their revised manuscript. Authors are encouraged (not required) to further revise their Materials and Methods if the SciScore is below 4. More information can be found here: https://rupress.org/jgp/pages/submission-guidelines#sciscore.

Please note, JGP now requires authors to submit Source Data used to generate figures containing gels and Western blots with all revised manuscripts (when applicable). This Source Data consists of fully uncropped and unprocessed images for each gel/blot displayed in the main and supplemental figures. If your paper includes cropped gel and/or blot images, please be sure to provide one Source Data file for each figure that contains gels and/or blots along with your revised manuscript files. File names for Source Data figures should be alphanumeric without any spaces or special characters (i.e., SourceDataF#, where F# refers

to the associated main figure number or SourceDataFS# for those associated with Supplementary figures). The lanes of the gels/blots should be labeled as they are in the associated figure, the place where cropping was applied should be marked (with a box), and molecular weight/size standards should be labeled wherever possible.
Source Data files will be made available to reviewers during evaluation of revised manuscripts and, if your paper is eventually published in JGP, the files will be directly linked to specific figures in the published article.

Source Data Figures should be provided as individual PDF files (one file per figure). Authors should endeavor to retain a minimum resolution of 300 dpi or pixels per inch. Please review our instructions for export from Photoshop, Illustrator, and PowerPoint here: https://rupress.org/jgp/pages/submission-guidelines#revised

Whilst you are revising your manuscript, we ask that you consider whether you have any artwork that might be suitable for the cover of JGP. Microscopy images are particularly good for cover artwork, but other types of image can be very effective, so we encourage you to be creative. Please don't restrict yourself to images from the paper; an image that is relevant to the work described would be just as suitable. Images should be a minimum resolution of 300 dpi. To see recent examples, visit the following page and click on 'Show covers? Yes': https://jgp.rupress.org/content/by/year)

Thank you for submitting your interesting research to JGP.

Please submit your revised manuscript, and any associated files, via this link:
Link Not Available

Sincerely,

Crina Nimigean, Ph.D.
On behalf of Journal of General Physiology

Journal of General Physiology's mission is to publish mechanistic and quantitative molecular and cellular physiology of the highest quality; to provide a best-in-class author experience; and to nurture future generations of independent researchers.

Reviewer #1 (Comments to the Authors):

The authors present a combined experimental/computational study characterizing a putative open state of the pentameric ligand-gated ion channel ELIC featuring a lateral ion conduction pathway through fenestrations that open in the simulations. Mutations along the lateral permeation pathway show modified currents in experiments, validating the simulation findings. Overall the study is of high quality, well written and of general interest. I have only a few minor points that should be addressed in a revised manuscript.

First, the correspondence between experimental and simulated currents is encouraging, but there are many considerations such as the lack of explicit electronic polarizability, that typically limit the quantitative agreement between simulated and measured currents. The manuscript would benefit from a discussion of these effects and a discussion on whether or not the obtained agreement in this case is perhaps in part somewhat fortuitous.

Second, although the ABF simulations are nicely supportive of the other simulation results, I do not quite get the motivation for carrying out the ABF simulations. Don't the computational electrophysiology simulations already provide a probability for the different pathways, including the presence of barriers along the way, via their Boltzmann weights? So what is the added value of the ABF simulations, other than the fact that they inform on the situation at equiibrium, so 0 mV?

Finally, the ABF PMFs (Fig. 4) are presented without error bars. It would be informative to indicate the statistical error.

Reviewer #2 (Comments to the Authors):

SUMMARY

In this study the authors use a combination of computational electrophysiology with Molecular Dynamics simulations as well as experimental single-channel recordings to elucidate the importance of lateral fenestrations in the extracellular vestibule to ion conductance in the bacterial pentameric ligand-gated ion channel, ELIC. The manuscript is reasonably well-written. In particular, the authors are to be commended in their aim to match simulations with experimental data in order gain a better understanding of the functional state of a solved ELIC structure.

GENERAL COMMENTS

- The single-channel data is fine. If anything, the authors should show more of it (see comments below).

- Given that the conductance from single-channel recordings and computational ephys agree reasonably well, does this mean that the MD equilibrated structure is the actual structure of the open channel? No! This is not necessarily so. As long as the 'rate-limiting step'/highest energy barrier is the same, the inferred conductance will be the same. This is reassuring, but not direct evidence that this is a physiologically-relevant open structure.

SPECIFIC COMMENTS

- To facilitate review, submitted manuscripts should have numbered lines.

- Introduction: "Perhaps the biggest drawback to this approach is that it ascribes a static structure to a dynamic functional state." What do you mean by this? How does this fit in this paragraph about pore profile? This should be clarified.

- When referring to computational electrophysiology you say "These methods allow direct comparison between the structural data and functional data, increasing confidence in assigned functional states." I agree that this comparison is better than doing nothing, but there are limitations to the accuracy of computational electrophysiology when compared to experiments (cellular or membrane complexity, other proteins, solutes, etc.) These are not mentioned, and I think it would strengthen your work to get out in front of these. The way it is written now, makes it sound as if computational electrophysiology is expected to perfectly match experimental electrophysiology... Is this fair?

- I understand that it is impractical to simulate 'ad infinitum', but can you really claim equilibration/stabilization of the backbone is complete at 500-600ns? At the end of your simulation the RMSD of both the TMD and M2 look to be creeping up a bit... Could this be a further structural rearrangement/equilibration? 1 us is still a relatively short timescale for these proteins. Do you need to claim equilibration/stabilization is reached? If not, don't.

- Please provide a rationale for why you chose the two specific frames you did from your production run. Currently this is not clear.

- Propylamine vs. Cysteamine - should indicate that they have the same IV behaviour (if not show this, at least find a reference). Make it clear what was done with what, and unitary conductance should be the same for both agonists.

- Along the same lines, POPC membranes were used in the MD, while POPC/POPG/POPE membranes were used for the experiments. Make clear whether or not this is something to be concerned about, because it is an obvious difference? For the 1us-long simulation could the lack of POPG or POPE make a difference? This limitation should be explicitly mentioned and addressed.

- While I like the Introduction as it clearly spells out some of the issues with interpreting structures, many of the raised issues are not dealt with explicitly in the current work. To tighten the story the introduction should be focussed on the issues specifically addressed.

- "The narrowest point is still located at E230 (a conserved ring of glutamates at the -1' position of the M2 helix) with an average radius of 2.4{plus minus}0.3A. This makes the channel still wide enough to conduct Na+ ions even after equilibration." Are you referring to a dehydrated or (partially) hydrated Na+ ion here? This should be made clear.

- "The hydrophobic residues at 9' and 16', L240 and F247, respectively, are the activation gates of ELIC based on the resting structure. The pore diameter at these residues is wide open in the ELIC5 open-channel structure, and there is a slight narrowing after equilibration of the simulation." Based on the MD Refined profile the narrowing of the pore is not 'slight', it is between 2-4A at these positions. In any case, 'slight' is a relative/subjective term and thus imprecise and vague. Be precise and quantitative. State the actual difference you observe.

- "While these are necessary conditions for the physiologic open-channel state, they are not sufficient. Further evidence is needed to demonstrate that sodium conduction through the channel matches the experimental value." This is a rate measurement (sodium conduction). Could be clearer and say something like "Further evidence is needed to demonstrate that the rate of sodium conduction through the channel matches the experimental value."

- While IV curves are shown for both WT and ELIC5 it would be nice to also see some single-channel traces for ELIC5, which is the main focus if this paper.

- The single channel data for measuring amplitudes is nice. It would be useful to present more than +/-100 mV data to help

illustrate how the I/V plot in Figure 2, panel C was produced.

- Figure 2, panels D and E: Are these necessary? Especially since the rectification of GlyR follows the opposite trend. My opinion is that discussion of the other channels should be relegated to the SI, and the generality of the findings should be discussed in the Discussion. Simulation on these additional channels detracts from the current story as is, and as presented does not convince me of the generality of the findings as applied to other pLGICs.

The authors need to provide a better rationale for including the computational electrophysiology of other channels, or relegate it to the Supplemental Information. For example, was the Alpha7 simulated to show that the double membrane approach provides the same results as fixed electric field? Unclear.

- Along the same lines as above... "These results demonstrate that the behavior observed in cryo-EM ELIC5 are not anomalous, but a trend observed across cryo-EM open-channel structures of pLGICs." This seems unnecessarily bold. I would recommend focussing the story on the interesting findings of ELIC here (lateral fenestrations, etc.). Leave the other computational electrophysiology for a more comprehensive detailed study that can better advocate for the generalizability of the findings.

- "Notably, the lateral pathway elucidated here for ELIC is similar to the one recently described for α1 GlyR (Cerdan et al., 2022), but has not been described in other pLGICs to date." Not entirely true. I think it is fair to say that Unwin hinted at these in his original, 20 year old now, model of the Torpedo nAChR at 4.5 A. See excerpt from Unwin's 2005 JMB paper below:

"The narrow interstitial spaces between the contact areas on the subunit interfaces are of special interest because they provide pathways (or potential pathways) for diffusing ions. Most of them are lined by polar or negatively charged side-chains and would therefore be selectively permeable to cations. THEY OCCUR ON BOTH SIDES OF THE MEMBRANE CLOSE TO THE MEMBRANE SURFACES. However, the largest open spaces are on the intracellular side, between neighbouring MA helices, and the surfaces framing each of these contain several negative charges (Figure 7)."

- Figure 7: It would be nice to also have recordings of the WT shown in this figure, and at the same scale, for reference.

- "Therefore, it seems prudent that all open-channel structures of pLGICs should be probed with MD simulations, which allow solvation, ions, and physiologic temperature, when examining structure-function relationships." This seems a little bold, and again fails to recognize the limitations of MD. Basically it sounds as though you are calling out structural biologists for not simulating their structures because of the limitations of static structures solved under conditions that are not physiologically relevant... At the same time you are not acknowledging that likewise, the MD simulations are not performed under physiological conditions, and thus are not perfect.

I agree that more data is better... But be more measured. I guess, is it necessary to say this, and what exactly are you trying to say? I'm not sure it is fair to say that equilibration, or even 'long' simulations, by MD makes these structures physiologically relevant, or even more physiologically relevant, nor does having their calculated computational conductance matching their actual conductance... Perhaps it is necessary, but not sufficient.

- Page 12, first paragraph of results "Several examples of purported open channel structures..." This sentence is grammatically incorrect. Portion of the sentence before "however" needs to be a complete clause.

- Figure 2 caption title - typo, "cyo" should be "cryo"

- Should be made clearer/emphasized more in the text that mutations in figure 7 were made in the WT background.

- Figure 3 "subunits removed for clarity" - how many and which should be stated in the figure caption

----

Specifically regarding the MD Methods:

- Please specify why the N-term was acetylated while the C-term was left uncapped in preparation of the MD simulation system.

- Specify the distance between the receptor and the periodic boundaries when using the reported lipid bilayer dimensions. (i.e. the rationale for the chosen lipid bilayer dimensions.)

- "Each propylamine was kept in its binding site throughout the full simulation via a weak (k = 0.25kcal/mol/A^2) bond between the terminal quaternary ammonium of propylamine and the δ-carbon of residues E77 and E131." Consider repharasing to avoid confusion. Were two weak bonds imposed (i.e., two distance restraints imposed? Quaternary amine--E77(Cδ) and Quaternary amine--E131(Cδ)?

- The methods specify ligands, ligand parametrization, initial system dimensions, and atom count used for the ELIC5 simulation system, but there is no mention of these in the methods for the alpha7 and GlyR simulation systems? Address this. These details are less important (lipid bilayer dimensions and atom count) or not important (ligand and parameters) for alpha7 and GlyR since no simulation/production run was performed for them. Indeed, according to methods: For ELIC5, computational Ephys was performed starting from (1) the last frame of a 50 ns equilibration run; and (2) two simulation frames selected once RMSD has stabilized in the 1 us production run. For alpha7 and GlyR, computational Ephys was performed starting from the last frame of a 50 ns equilibration run.

- Is there a rationale for studying the ELIC5 simulation system at 310 K, while studying alpha7 and GlyR simulation systems at 300 K? Could this affect comparability of their computational EPhys data? I assume 300 K was chosen for alpha7 to allow for comparison with the Zhang et al. (2022) computational Ephys with the double-membrane approach, as they used 300 K. This is not mentioned in the present manuscript.

Reviewer #3 (Comments to the Authors):

The manuscript by Arcario et al. addresses an important and timely problem for the ion channel field in general: how to interpret "open" cryo-EM structures that appear conductive, but can show non trivial effects in MD simulations under applied voltage. The authors focus on a member of the pLGIC family and combine unbiased MD, ABF free-energy calculations, computational electrophysiology, and single-channel recordings with targeted mutagenesis to address an apparent contradiction between simulations and experiment regarding anomalous rectification observed in initial computational electrophysiology simulations.

In my opinion, the central point of the manuscript is appealing: relaxation/expansion of the Extracellular Domian (ECD) enables/enhances a lateral ECD fenestration pathway, that becomes dominant and reconciles the large outward rectification predicted from the cryo-EM snapshot with the near-ohmic behavior seen experimentally. However, several issues currently limit my confidence in this claim and should be addressed before I can recommend this manuscript for publication.

Major points:

The authors base their model on a single 1us long trajectory, that showed that spontaneous ECD widening / opening of the lateral pathway. I'd like to see that this is happening reproducibly across at least ~3 replicas (or show it by some other means). They do not need to be 1us long necessarily. A short discussion why/how this widening occurs would be a great addition too.

The structures with substantial lateral opening improve rectification and bring it closer to experiments, but simulations still show too high conductances as compared to experimental estimates. Some further analysis why this is the case would be helpful here. Please consider reporting conductance uncertainties across replicas (not just linear fit, clarify the fitting procedure (are the lines forces to go through the origin?) and given high conductances at 250mV, consider running shorter simulations at lower voltages, for any possible non-linear behaviour traces.

The authors decided to use harmonic restraints in all computational electrophysiology simulations. The observed conductance then will heavily depend on the chosen initial frame. Ideally one would like to see how conductances are shaped in the absence of restrains, and how the dynamics of both pathways affect the final conductance and rectification values. If this is not feasible, the authors should clearly indicate this in the discussion, that predicted conductances are method/snapshot dependent and do not include the full dynamics of the channel

The ABF maps indicate strong cation stabilization at acidic ECD regions, and the manuscript discusses known Ca^{2+}/Ba^{2+} effects. Yet the experimental solutions include Ba^{2+}, which can screen/occupy acidic clusters and plausibly modulate entry routes and rectification. Is it possible to perform some of these recordings without Ba^{2+} ions? If not feasible, the potential effect of divalent ions on the proposed lateral pathway should be discussed.

Minor points:
The manuscript appears to list the ELIC5 mutations in different directions in different places (i.e., WT->ELIC5 vs ELIC5->WT). Please provide a clear table with the numbering scheme and clear description of WT -> ELIC5 residues.
Lipid composition: pure POPC in simulations and 2:1:1 POPC:POPE:POPG in experiments. Please add short discussion if lipid composition can affect the conclusions about ion pathway preference

A schematic picture showing 'apical pathway' and 'lateral pathway/fenestration' would help to grasp the idea early

Several panels lack error bars (PMFs, number of permeation events [you can consider Poisson distribution]) etc. Please add them.

"computational electrophysiology" is the name of the GROMACS module for simulating ion channels in a double membrane system (as introduced by Kutzner et al., BJ 2011). To avoid confusion, I suggest that the authors replace this term by "in silico

electrophysiology" or "applied voltage simulations" in their manuscript.

Reviewer #1 (Comments to the Authors):

The authors present a combined experimental/computational study characterizing a putative open state of the pentameric ligand-gated ion channel ELIC featuring a lateral ion conduction pathway through fenestrations that open in the simulations. Mutations along the lateral permeation pathway show modified currents in experiments, validating the simulation findings. Overall the study is of high quality, well written and of general interest. I have only a few minor points that should be addressed in a revised manuscript.

First, the correspondence between experimental and simulated currents is encouraging, but there are many considerations such as the lack of explicit electronic polarizability, that typically limit the quantitative agreement between simulated and measured currents. The manuscript would benefit from a discussion of these effects and a discussion on whether or not the obtained agreement in this case is perhaps in part somewhat fortuitous.

We appreciate the reviewer's suggestion. We have included a discussion on the potential effects of polarizable force fields in the manuscript. We do not believe the agreement is fortuitous in this manuscript as many pieces, including the ABF and electrophysiology data seem to support the findings of the lateral fenestration being open.

Second, although the ABF simulations are nicely supportive of the other simulation results, I do not quite get the motivation for carrying out the ABF simulations. Don't the computational electrophysiology simulations already provide a probability for the different pathways, including the presence of barriers along the way, via their Boltzmann weights? So what is the added value of the ABF simulations, other than the fact that they inform on the situation at equiibrium, so 0 mV?

The reviewer is correct in their assertion that a rough free energy surface could be constructed from the probability density of ions within the channel at each potential and could indicate potential barriers. We were concerned, however, that there were not enough ion crossing events and not enough ion density within the channel itself to accurately reflect any free energy barriers/wells. Therefore, we performed the ABF calculation to explicitly determine the free energy surface of ion translocation within the channel for further analysis. We have rewritten the section on ABF to make this more apparent.

Finally, the ABF PMFs (Fig. 4) are presented without error bars. It would be informative to indicate the statistical error.

We have addressed this in the revisions. Calculating the statistical error for ABF profiles can be quite difficult and the best method to estimate statistical error is replicating the ABF calculation. Given that this calculation is multiple dimensions across the entirety of the channel, it would be prohibitively costly to calculate the statistical error with replicates. Here, however, we have estimated the upper bound of the error for each sampling window using the heterogeneity of sampling within each window. With infinite time for each sampling window, all space within the window would be sampled evenly. The sampling heterogeneity gives an estimate of how far from an evenly sampled free energy surface we have. Therefore, this would provide the worst-case scenario for the error. This information is now provided as Fig. S4. We have also included the convergence data for each window as Fig. S5.

Reviewer #2 (Comments to the Authors):

SUMMARY

In this study the authors use a combination of computational electrophysiology with Molecular Dynamics simulations as well as experimental single-channel recordings to elucidate the importance of lateral fenestrations in the extracellular vestibule to ion conductance in the bacterial pentameric ligand-gated ion channel, ELIC. The manuscript is reasonably well-written. In particular, the authors are to be commended in their aim to match simulations with experimental data in order gain a better understanding of the functional state of a solved ELIC structure.

GENERAL COMMENTS

- The single-channel data is fine. If anything, the authors should show more of it (see comments below).

- Given that the conductance from single-channel recordings and computational ephys agree reasonably well, does this mean that the MD equilibrated structure is the actual structure of the open channel? No! This is not necessarily so. As long as the 'rate-limiting step'/highest energy barrier is the same, the inferred conductance will be the same. This is reassuring, but not direct evidence that this is a physiologically-

relevant open structure.
We agree and have made the language through more tempered, emphasizing structural changes that improve agreement between structures and functional data.

SPECIFIC COMMENTS

- To facilitate review, submitted manuscripts should have numbered lines.

- Introduction: "Perhaps the biggest drawback to this approach is that it ascribes a static structure to a dynamic functional state." What do you mean by this? How does this fit in this paragraph about pore profile? This should be clarified.
We have rewritten this sentence to be more straightforward. Our argument is that assigning what is an inherently dynamic functional state (closed, open, desensitized) from a single metric (TMD pore radius) gathered from a spatially-averaged structure can be problematic. We certainly believe that MD can circumvent some of these issues with more dynamic metrics and time-averaged structures.

- When referring to computational electrophysiology you say "These methods allow direct comparison between the structural data and functional data, increasing confidence in assigned functional states." I agree that this comparison is better than doing nothing, but there are limitations to the accuracy of computational electrophysiology when compared to experiments (cellular or membrane complexity, other proteins, solutes, etc.) These are not mentioned, and I think it would strengthen your work to get out in front of these. The way it is written now, makes it sound as if computational electrophysiology is expected to perfectly match experimental electrophysiology... Is this fair?

We agree it is unfair to expect computational electrophysiology results to exactly match experimental data with differences in experimental design and limited opportunities to make experimental and computational design match exactly. We have added several sentences to the introduction explicitly stating this and providing a series of possible differences between experimental and computational set ups. We believe this frames the problem and solution in a more measured manner.

- I understand that it is impractical to simulate 'ad infinitum', but can you really claim equilibration/stabilization of the backbone is complete at 500-600ns? At the

end of your simulation the RMSD of both the TMD and M2 look to be creeping up a bit… Could this be a further structural rearrangement/equilibration? 1 us is still a relatively short timescale for these proteins. Do you need to claim equilibration/stabilization is reached? If not, don't.

We have changed the language to a "locally-stable state". It is unknown whether this is an "equilibrium" structure, but general language in the field states structures as equilibrated once they have reached a local structural minimum, which is the convention we were using.

- Please provide a rationale for why you chose the two specific frames you did from your production run. Currently this is not clear.

We have added additional description of why we chose the frames we did. We noted through our simulation two large structural changes in the ECD, namely the widening of a lateral fenestration as well as expansion of the two rings of basic residues along the apical pathway (R65/K90). Since the computational electrophysiology showed that widening of the lateral pathway significantly improved rectification and agreement with functional data (structure 2), but there was still rectification and discrepancy with experimental data we looked to see if choosing a structure with a narrower apical opening would further improve agreement, based on the rationale that this is not the main conduction pathway (based on ABF results). Structure 1 has a widened lateral pathway and narrower apical opening, and indeed, does improve rectification of the channel as well as improving agreement with experimental conductances. This discussion has been included in the revision.

- Propylamine vs. Cysteamine - should indicate that they have the same IV behaviour (if not show this, at least find a reference). Make it clear what was done with what, and unitary conductance should be the same for both agonists.

This is an excellent question and we appreciate the reviewer raising it. It has been demonstrated previously that cysteamine and propylamine are equivalent agonists, supporting equivalent open probability. In fact, for ELIC, authors will use propylamine and cysteamine interchangeably due to their similarity for both macroscopic and single channel properties. We have included discussion regarding this in the Methods section "Modeling and Equilibration of the ELIC5 Open State for Molecular Dynamics Simulation". In addition, we have obtained single-channel currents for WT ELIC activated by propylamine and added those to Figure 2. The single-channel conductance for WT ELIC activated by propylamine (86.6) is statistically the same as the single-channel conductance of WT ELIC activated by

cysteamine (88.7). Data regarding the lack of rectification observed with propylamine activation has also been added to Table 2.

- Along the same lines, POPC membranes were used in the MD, while POPC/POPG/POPE membranes were used for the experiments. Make clear whether or not this is something to be concerned about, because it is an obvious difference? For the 1us-long simulation could the lack of POPG or POPE make a difference? This limitation should be explicitly mentioned and addressed.

We have added a discussion on why we chose POPC as the solvating lipid for the computational studies and anticipated effects. Namely, the other computational electrophysiology studies that we are comparing to (Cerdan, 2018, 2020 and Noviello 2021) use POPC as the solvating lipid and so it makes sense to do the same to allow direct comparison. We know from previous experiments in our lab that the 2:1:1 POPC:POPE:POPG ratio is a maximally activating condition, but it is known that POPC-only membranes do support activation of ELIC by propylamine (Kumar 2020). We have added this discussion to the Methods section "Modeling and Equilibration of the ELIC5 Open State for Molecular Dynamics Simulation".

- While I like the Introduction as it clearly spells out some of the issues with interpreting structures, many of the raised issues are not dealt with explicitly in the current work. To tighten the story the introduction should be focussed on the issues specifically addressed.

We have revised the Introduction section to be more focused as well as balanced. We hope this improves the readability and lays out the problem we are addressing in this manuscript more succinctly.

- "The narrowest point is still located at E230 (a conserved ring of glutamates at the -1' position of the M2 helix) with an average radius of 2.4{plus minus}0.3A. This makes the channel still wide enough to conduct Na+ ions even after equilibration." Are you referring to a dehydrated or (partially) hydrated Na+ ion here? This should be made clear.

We have updated the language of this sentence to be more precise in the size of sodium ions and what will fit through the narrowest portion of the channel (sterically).

- "The hydrophobic residues at 9' and 16', L240 and F247, respectively, are the activation gates of ELIC based on the resting structure. The pore diameter at these residues is wide open in the ELIC5 open-channel structure, and there is a slight narrowing after equilibration of the simulation." Based on the MD Refined profile the narrowing of the pore is not 'slight', it is between 2-4A at these positions. In any case, 'slight' is a relative/subjective term and thus imprecise and vague. Be precise and quantitative. State the actual difference you observe.

We appreciate the reviewer's comments on this. We have removed qualitative descriptors of the pore radius dynamics. We have added additional quantitative descriptors to this section to help readers observe how dynamic these channels are.

- "While these are necessary conditions for the physiologic open-channel state, they are not sufficient. Further evidence is needed to demonstrate that sodium conduction through the channel matches the experimental value." This is a rate measurement (sodium conduction). Could be clearer and say something like "Further evidence is needed to demonstrate that the rate of sodium conduction through the channel matches the experimental value."

We appreciate the suggestion for clarity and have made this change in the manuscript. Along with the other changes, we hope this greatly improves the clarity of the manuscript.

- While IV curves are shown for both WT and ELIC5 it would be nice to also see some single-channel traces for ELIC5, which is the main focus if this paper.

We agree with the reviewer here and have added traces for ELIC5 to Figure 2 for completeness and for clarity.

- The single channel data for measuring amplitudes is nice. It would be useful to present more than +/-100 mV data to help illustrate how the I/V plot in Figure 2, panel C was produced.

While we appreciate the reviewer's favorable opinion of the single-channel data we feel that the sample traces at +/-100 mV adequately illustrate the quality of the single channel currents, and that the method of determining the I-V curve is well-described. Current traces at other voltages have a very similar appearance only with different current amplitudes. To maintain readability, we do not think it is necessary to include more single-channel current

traces to this already dense figure.

- Figure 2, panels D and E: Are these necessary? Especially since the rectification of GlyR follows the opposite trend. My opinion is that discussion of the other channels should be relegated to the SI, and the generality of the findings should be discussed in the Discussion. Simulation on these additional channels detracts from the current story as is, and as presented does not convince me of the generality of the findings as applied to other pLGICs.

The authors need to provide a better rationale for including the computational electrophysiology of other channels, or relegate it to the Supplemental Information. For example, was the Alpha7 simulated to show that the double membrane approach provides the same results as fixed electric field? Unclear.

- Along the same lines as above... "These results demonstrate that the behavior observed in cryo-EM ELIC5 are not anomalous, but a trend observed across cryo-EM open-channel structures of pLGICs." This seems unnecessarily bold. I would recommend focussing the story on the interesting findings of ELIC here (lateral fenestrations, etc.). Leave the other computational electrophysiology for a more comprehensive detailed study that can better advocate for the generalizability of the findings.

We believe these findings are important to the overall discussion of cryo-EM open-channel structures reported here. We also believe it is important to disseminate the results we have regarding the alpha1 GlyR and alpha7 nAChR so others are aware of potential issues with these open channel structures. We do recognize, however, that our initial language was overly generalized and so have revised the language to focus specifically on the constructs we have tested here.

- "Notably, the lateral pathway elucidated here for ELIC is similar to the one recently described for α1 GlyR (Cerdan et al., 2022), but has not been described in other pLGICs to date." Not entirely true. I think it is fair to say that Unwin hinted at these in his original, 20 year old now, model of the Torpedo nAChR at 4.5 A. See excerpt from Unwin's 2005 JMB paper below:

"The narrow interstitial spaces between the contact areas on the subunit interfaces are of special interest because they provide pathways (or potential pathways) for

diffusing ions. Most of them are lined by polar or negatively charged side-chains and would therefore be selectively permeable to cations. THEY OCCUR ON BOTH SIDES OF THE MEMBRANE CLOSE TO THE MEMBRANE SURFACES. However, the largest open spaces are on the intracellular side, between neighbouring MA helices, and the surfaces framing each of these contain several negative charges (Figure 7)."

Thank you for mentioning this reference. We were unaware of this and have included the reference in our discussion regarding this pathway in anion- and cation-selective channels.

- Figure 7: It would be nice to also have recordings of the WT shown in this figure, and at the same scale, for reference.

We have added wild-type recordings to this figure.

- "Therefore, it seems prudent that all open-channel structures of pLGICs should be probed with MD simulations, which allow solvation, ions, and physiologic temperature, when examining structure-function relationships." This seems a little bold, and again fails to recognize the limitations of MD. Basically it sounds as though you are calling out structural biologists for not simulating their structures because of the limitations of static structures solved under conditions that are not physiologically relevant... At the same time you are not acknowledging that likewise, the MD simulations are not performed under physiological conditions, and thus are not perfect.

I agree that more data is better... But be more measured. I guess, is it necessary to say this, and what exactly are you trying to say? I'm not sure it is fair to say that equilibration, or even 'long' simulations, by MD makes these structures physiologically relevant, or even more physiologically relevant, nor does having their calculated computational conductance matching their actual conductance... Perhaps it is necessary, but not sufficient.

We have softened the language of this closing line (in the Discussion section). Our intention was to promote MD as a tool to further examine structures critically and possibly learn new information and mechanistic insight into how conformation and function are related. We hope our revisions make this sentiment more clear.

- Page 12, first paragraph of results "Several examples of purported open channel structures..." This sentence is grammatically incorrect. Portion of the sentence before

"however" needs to be a complete clause.
We have revised this sentence and the sentences surrounding this to make our point more clear.

- Figure 2 caption title - typo, "cyo" should be "cryo"
This typo has been fixed for the revision.

- Should be made clearer/emphasized more in the text that mutations in figure 7 were made in the WT background.
We have added this to the figure caption as well as into the main body of the manuscript.

- Figure 3 "subunits removed for clarity" - how many and which should be stated in the figure caption
We have added additional description on which subunit was removed to help orient the reader.

Specifically regarding the MD Methods:

- Please specify why the N-term was acetylated while the C-term was left uncapped in preparation of the MD simulation system.
This has been addressed in the resubmission in the sections “Modeling and Equilibration of the ELIC5 Open State for Molecular Dynamics Simulation” and “Modeling of the α7 nAChR and α1 GlyR Open-Channel Structures”.

- Specify the distance between the receptor and the periodic boundaries when using the reported lipid bilayer dimensions. (i.e. the rationale for the chosen lipid bilayer dimensions.)
We have added the minimum distance between periodic images for all simulation systems to the sections “Modeling and Equilibration of the ELIC5 Open State for Molecular Dynamics Simulation” and “Modeling of the α7 nAChR and α1 GlyR Open-Channel Structures”.

- "Each propylamine was kept in its binding site throughout the full simulation via a weak (k = 0.25kcal/mol/A^2) bond between the terminal quaternary

ammonium of propylamine and the δ-carbon of residues E77 and E131." Consider repharasing to avoid confusion. Were two weak bonds imposed (i.e., two distance restraints imposed? Quaternary amine--E77(Cδ) and Quaternary amine--E131(Cδ)?
We have been much more descriptive of these weak bonds in the section "Modeling and Equilibration of the ELIC5 Open State for Molecular Dynamics Simulation" and hope this improves the clarity of these restraints.

- The methods specify ligands, ligand parametrization, initial system dimensions, and atom count used for the ELIC5 simulation system, but there is no mention of these in the methods for the alpha7 and GlyR simulation systems? Address this. These details are less important (lipid bilayer dimensions and atom count) or not important (ligand and parameters) for alpha7 and GlyR since no simulation/production run was performed for them. Indeed, according to methods: For ELIC5, computational Ephys was performed starting from (1) the last frame of a 50 ns equilibration run; and (2) two simulation frames selected once RMSD has stabilized in the 1 us production run. For alpha7 and GlyR, computational Ephys was performed starting from the last frame of a 50 ns equilibration run.

We have added additional details to the Methods section "Modeling of the α7 nAChR and α1 GlyR Open-Channel Structures" regarding system size and composition for the GlyR and nAChR systems to be better in line with the ELIC5 system description. Since the backbone was restrained for the GlyR and nAChR systems, no ligand was added to the orthosteric site. This has been made clear in the revision.

- Is there a rationale for studying the ELIC5 simulation system at 310 K, while studying alpha7 and GlyR simulation systems at 300 K? Could this affect comparability of their computational EPhys data? I assume 300 K was chosen for alpha7 to allow for comparison with the Zhang et al. (2022) computational Ephys with the double-membrane approach, as they used 300 K. This is not mentioned in the present manuscript.
Yes, these temperatures were chosen to allow direct comparison between this study and others. This has been explicitly stated in the Methods section.

Reviewer #3 (Comments to the Authors):

The manuscript by Arcario et al. addresses an important and timely problem for the ion channel field in general: how to interpret "open" cryo-EM structures that appear conductive, but can show non trivial effects in MD simulations under applied voltage. The authors focus on a member of the pLGIC family and combine unbiased MD, ABF free-energy calculations, computational electrophysiology, and single-channel recordings with targeted mutagenesis to address an apparent contradiction between simulations and experiment regarding anomalous rectification observed in initial computational electrophysiology simulations.

In my opinion, the central point of the manuscript is appealing: relaxation/expansion of the Extracellular Domian (ECD) enables/enhances a lateral ECD fenestration pathway, that becomes dominant and reconciles the large outward rectification predicted from the cryo-EM snapshot with the near-ohmic behavior seen experimentally. However, several issues currently limit my confidence in this claim and should be addressed before I can recommend this manuscript for publication.

Major points:

The authors base their model on a single 1us long trajectory, that showed that spontaneous ECD widening / opening of the lateral pathway. I'd like to see that this is happening reproducibly across at least ~3 replicas (or show it by some other means). They do not need to be 1us long necessarily. A short discussion why/how this widening occurs would be a great addition too.

We thank the review for their insightful suggestion. We have increased the number of replicates to 4 all running 1 microsecond, totaling 4 microseconds of unbiased simulation. We show that the lateral pathway widens in each of the replicates to varying degrees, but always wide enough to accommodate a sodium ion. As for a short discussion on why the lateral pathway widens compared to the cryo-EM structure, we could speculate multiple reasons why, but it would be beyond the data presented to ascribe a cause for the lateral widening compared to the cryo-EM structure.

The structures with substantial lateral opening improve rectification and bring it closer to experiments, but simulations still show too high conductances as compared to experimental estimates. Some further analysis why this is the case would be helpful here. Please consider reporting conductance uncertainties across replicas (not just linear fit, clarify the fitting procedure (are the lines forces to go through the origin?)

and given high conductances at 250mV, consider running shorter simulations at lower voltages, for any possible non-linear behaviour traces.

We have clarified our fitting procedures throughout the manuscript. In the case of the ELIC5 TMD-only, alpha7 nAChR, and alpha1 GlyR, we have enforced that the line goes through the origin as we do not have any data for this transmembrane potential and make the assumption that the current is 0 pA in a symmetric salt solution at 0 mV. For all other applied electric field simulations, we have not made this assumption and used the data presented in the manuscript for the linear fit. Uncertainties for the conductances are reported in the figure as the error associated with the linear fit as well as in Table 2.

Moreover, we have ran additional simulations of Structure 1 at +150 mV and -150 mV (i.e., lower voltages) to determine if there is any non-linearity in the applied field simulations. As shown from the data, there is no non-linearity involved in this process. We have expanded discussion on the remaining rectification after MD relaxation as well.

The authors decided to use harmonic restraints in all computational electrophysiology simulations. The observed conductance then will heavily depend on the chosen initial frame. Ideally one would like to see how conductances are shaped in the absence of restrains, and how the dynamics of both pathways affect the final conductance and rectification values. If this is not feasible, the authors should clearly indicate this in the discussion, that predicted conductances are method/snapshot dependent and do not include the full dynamics of the channel

We agree that using restraints on the protein backbone will influence the measured conductance. We chose to use these restraints so we could ascribe a conductance to a well-defined channel structure. Certainly, it would be expected that the protein would change conformations in the combined presence of a high (250 mV) transmembrane potential and the absence of backbone harmonic restraints. While performing another full set of simulations without restraints is prohibitive for this study, we have added to the Discussion that computational electrophysiology results are dependent on the frames chosen.

The ABF maps indicate strong cation stabilization at acidic ECD regions, and the manuscript discusses known Ca^{2+}/Ba^{2+} effects. Yet the experimental solutions include Ba^{2+}, which can screen/occupy acidic clusters and plausibly modulate entry routes and rectification. Is it possible to perform some of these recordings without Ba^{2+} ions? If not feasible, the potential effect of divalent ions on the proposed lateral pathway should be discussed.

While Ba2+ has been shown to block the channel, we are using a very small concentration (0.5 mM) to promote patch formation on the pipette. According to work from Raimund Dutzler (Zimmermann, et al 2012), this concentration of Ba2+ should have little to no effect on activation of the channel at the concentration of cysteamine used in the manuscript and has little to no effect on single channel currents at such a low concentration.

Minor points:

The manuscript appears to list the ELIC5 mutations in different directions in different places (i.e., WT->ELIC5 vs ELIC5->WT). Please provide a clear table with the numbering scheme and clear description of WT -> ELIC5 residues.

We apologize for the inconsistency. This has been edited in the revised manuscript. Now, all mutations are for WT->ELIC5.

Lipid composition: pure POPC in simulations and 2:1:1 POPC:POPE:POPG in experiments. Please add short discussion if lipid composition can affect the conclusions about ion pathway preference

We appreciate the thoughtful reading by the reviewer. We had added a discussion regarding the possible effects of the lipids in this manuscript (in the Discussion section).

A schematic picture showing 'apical pathway' and 'lateral pathway/fenestration' would help to grasp the idea early

Thank you for the excellent suggestion! We have added a schematic figure (Figure 8) to demonstrate the location of the apical pathway versus the lateral pathway and hope this helps in understanding the manuscript.

Several panels lack error bars (PMFs, number of permeation events [you can consider Poisson distribution]) etc. Please add them.

We have added additional error bars and estimates of error to measurements throughout the manuscript and have described how we have arrived at these error estimates.

"computational electrophysiology" is the name of the GROMACS module for simulating ion channels in a double membrane system (as introduced by Kutzner et al., BJ 2011). To avoid confusion, I suggest that the authors replace this term by "in silico electrophysiology" or "applied voltage simulations" in their manuscript.

While we acknowledge "computational electrophysiology" is a module name within the GROMACS ecosystem, many groups who have used this approach (applied field/applied voltage/applied electric field) have used the moniker "computational electrophysiology". We do not believe this causes confusion and have explicitly stated in the manuscript that we are using an applied field approach versus the double membrane approach of the "computational electrophysiology" model. In addition, we have added a schematic (Figure 2A) to emphasize the approach used in this manuscript.

Dr. Wayland W. L. Cheng
Washington University School of Medicine
Anesthesiology
660 S. Euclid Ave.
St. Louis, MO 63110

Re: 202513926R1

Dear Wayland,

I am pleased to let you know that your manuscript, titled "Characterization of an Open-Channel Structure and Lateral Conduction Pathway in the Cation-Selective Pentameric Ligand-Gated Ion Channel, ELIC" is scientifically acceptable for publication in Journal of General Physiology. Formal acceptance will follow when it is modified in accordance with the referees' remarks and our editorial policies.

Please note items that need attention are listed at the bottom of this email (under 'manuscript formatting checklist'). Please also be sure to include a letter addressing the reviewers' comments point-by-point (if applicable) and a copy of the text with alterations highlighted (boldfaced or underlined). Your manuscript should be a double-spaced MS Word file and include editable tables, if appropriate.

Lastly, JGP requires a data availability statement for all research article submissions. These statements will be published in the article directly above the Acknowledgments. The statement should address all data underlying the research presented in the manuscript. Please visit the JGP instructions for authors for guidelines and examples of statements at https://rupress.org/jgp/pages/editorial-policies#data-availability-statement.

Please submit your final files via this link:
Link Not Available

Thank you for choosing to publish your research in JGP and please feel free to contact me with any questions.

Sincerely,

Crina Nimigean, Ph.D.
On behalf of Journal of General Physiology

Journal of General Physiology's mission is to publish mechanistic and quantitative molecular and cellular physiology of the highest quality; to provide a best in class author experience; and to nurture future generations of independent researchers.

Manuscript formatting checklist:
- MS Word document of text needed (including editable tables)
- MS Word document of supplemental text needed, if applicable (including figure legends and editable tables)
- Brief Statement describing supplementary information needed, if applicable (in subsection at end of Materials & Methods)
- Please include a data availability statement preceding the Acknowledgments section. Please see https://rupress.org/jgp/pages/editorial-policies#data-availability-statement
- Figures created at sufficient resolution and in acceptable format (including supplemental if applicable). If working in Illustrator, we prefer .ai or .eps file format. If working in Photoshop please use 600dpi/1000dpi .tiff or .psd file format. Minimum resolution at estimated print size: Minimum resolution for all figures is 600 dpi. For figures that contain both photographs and line art or text, 600 dpi is highly recommended. Figures containing only black and white elements (line art, no color, and no gray) should be 1,000 dpi. Maximum figure size is 7 in wide x 9 in high (17.5 x 22.8 cm) at the correct resolution. https://jgp.rupress.org/fig-vid-guidelines
- Supplemental figures, if any, conforming to same guidelines as manuscript figures (noted above)
- If images resemble one from a prior publications, the author must seek permissions (to reproduce or adapt) from the original publisher. [You can resubmit your paper while waiting to hear back from the original publisher but please keep us updated]
- All authors must complete a disclosure form prior to acceptance. A link to complete the form has been sent to all coauthors. Please provide the editorial office with updated email addresses if necessary

---

Reviewer #1 (Comments to the Authors):

The authors have satisfactorily addressed my concerns.

Reviewer #2 (Comments to the Authors):

The manuscript is greatly improved, and authors have satisfactorily addressed my concerns.

Reviewer #3 (Comments to the Authors):

all my comments were sufficiently addressed, i recommend it for publication
